# Toward a Mature Science of Consciousness

**DOI:** 10.3389/fpsyg.2018.00693

**Published:** 2018-05-29

**Authors:** Wanja Wiese

**Affiliations:** Department of Philosophy, Johannes Gutenberg-Universität Mainz, Mainz, Germany

**Keywords:** consciousness, neurophenomenology, naturalized phenomenology, neural correlates of consciousness (NCCs), explanatory correlates of consciousness (ECCs), phenomenology, predictive processing, integrated information theory

## Abstract

In *Being No One*, Metzinger (2004[2003]) introduces an approach to the scientific study of consciousness that draws on theories and results from different disciplines, targeted at multiple levels of analysis. Descriptions and assumptions formulated at, for instance, the phenomenological, representationalist, and neurobiological levels of analysis provide different perspectives on the same phenomenon, which can ultimately yield necessary and sufficient conditions for applying the concept of phenomenal representation. In this way, the “method of interdisciplinary constraint satisfaction (MICS)” (as it has been called by Weisberg, 2005) promotes our understanding of consciousness. However, even more than a decade after the first publication of *Being No One*, we still lack a mature science of consciousness. This paper makes the following meta-theoretical contribution: It analyzes the hurdles an approach such as MICS has yet to overcome and discusses to what extent existing approaches solve the problems left open by MICS. Furthermore, it argues that a unifying theory of different features of consciousness is required to reach a mature science of consciousness.

## 1. Introduction

How far away is the science of consciousness from reaching a paradigmatic stage? One could argue that the science of consciousness is paradigmatic in the sense that we have well established sets of study paradigms, such as binocular rivalry, backward masking, continuous flash suppression, and many others. There are also first- and third-person *measures* of consciousness, the most common being verbal or non-verbal *report*, which has recently been complemented by proposals of “no-report paradigms” (see Tsuchiya et al., 2015, 2016a; Overgaard and Fazekas, 2016). However, there is reason to doubt that such measures operationalize the same phenomenon, since, as Irvine (2017) points out, “depending on what and how you measure, you get different answers, in both artificial and natural settings.” (Irvine, 2017, p. 101).

Empirically, this suggests that there is a lack of agreement on what the relevant phenomenon is that the science of consciousness is set out to study. Theoretically, the ongoing debate about phenomenal consciousness and access consciousness (see Cohen et al., 2016a,b; McClelland and Bayne, 2016), about levels of consciousness (see Bayne et al., 2016a,b; Fazekas and Overgaard, 2016), as well as about the distinction between conscious and unconscious perception (see Peters et al., 2017; Melnikoff and Bargh, 2018), further corroborates the view that there is no wide-spread consensus on what the relevant concept of consciousness is.

So there is a sense in which the science of consciousness is not paradigmatic, because one can argue that *consciousness* is not a unitary concept. Furthermore, ongoing discussions regarding the “hard problem” of consciousness (Chalmers, 1995)—Is it a real problem? Can materialist theories solve the problem? Can it be solved at all?—support the view that there is no consensus on what the fundamental puzzles in consciousness research are, as well as whether and how they can be solved.

Incidentally, in the 1969 postscript of *The Structure of Scientific Revolutions*, Thomas Kuhn points out that what characterizes a mature science is not typically just the possession of a *paradigm*, but of a particular type of paradigm: “What changes with the transition to maturity is not the presence of a paradigm, but rather its nature. Only after the change is normal puzzle-solving research possible.” (Kuhn, 1970, p. 179). Normal puzzle-solving research requires a consensus on what the relevant scientific puzzles are and how they can be solved (at least in principle). This suggests that the science of consciousness has not reached a mature stage yet (in Kuhn's sense), even if some scientific endeavors within the study of consciousness can be regarded as paradigmatic.

In this paper, I will argue that the conceptual framework set up in Thomas Metzinger's *Being No One* provides a path towards reaching a mature science of consciousness, although it will need a unifying theory to fully accomplish this goal. The core idea is this: A mature science of consciousness needs a well-defined target, picked out by a concept of consciousness that is non-controversial (because “consciousness” is not a technical term) and applies to at least most subjects of experience in ordinary states. Such a concept must involve characteristic (perhaps necessary) features of consciousness. Understanding these features will then lead to an understanding of consciousness.^1^ So far, this idea corresponds to the method pursued in Metzinger (2004[2003]), which Weisberg (2005) has called the “method of interdisciplinary constraint satisfaction (MICS).” MICS respects that the science of consciousness is multidisciplinary, without presupposing a fixed list of disciplines from which to expect relevant contributions. Correspondingly, MICS proposes to analyze features of consciousness on the phenomenological, representationalist, informational-computational, functional, and physical-neurobiological levels of descriptions, without presupposing that these are the only levels of description that are relevant (see Metzinger, 2004[2003], p. 110).

But there are two methodological integration problems not addressed by MICS: (1) How can analyses (operating at distinct levels of descriptions) of one and the same feature of consciousness be integrated with one another? (2) How can analyses of *different* features of consciousness be integrated in such a way that it becomes clear why they are features of a single phenomenon (as opposed to features of different types of consciousness)? These problems have to be solved to further advance the science of consciousness.

The paper is structured as follows. In section 2, I will summarize the key assumptions of Metzinger's MICS, illustrate the method using the example of global availability (section 2.1), and point out some shortcomings (section 2.2). One of the goals of MICS is to provide multi-disciplinary *descriptions* of features of consciousness that enable *explanations* of these features. Since we need more than just “intuitively coherent” descriptions at different levels, there must be a way to assess to what extent different descriptions match one another. I call this the *problem of matching descriptions*. A more specific problem that results from this I shall call the *problem of matching predicates*. In section 3, I will review existing proposals on how to solve these matching problems. The result will be that existing solutions are limited in scope. This suggests that different methods must be combined. Furthermore, consciousness is different from a mere bundle of features. I will argue that this will require an integrated account of different features, for instance, in terms of a single underlying computational principle (such as minimizing expected free energy, see Friston, 2018).

## 2. Thomas metzinger's MICS and the problem of consciousness

The question “What is consciousness?” can be interpreted in at least two ways: (1) It can be understood as a question that asks for the *explanandum*, which defines the target for the scientific study of consciousness. (2) Or it can be read as asking for the *explanans*, e.g., the neural mechanisms that underpin conscious experience. Answering the first question seems to be almost as difficult as answering the second question—which is only more puzzling given the fact that consciousness is not a rare phenomenon, but something we enjoy everyday, and something we are intimately familiar with (at least this is how it seems from the first-person perspective).

This suggests that consciousness cannot be studied in the same way as any ordinary natural phenomenon (such as water or the genetics of drosophila). Pessimistically, one may even suggest that consciousness is not a scientific concept, by arguing that there are not only two different types of access to consciousness (one direct, from the first-person perspective, the other indirect, from the third-person perspective), but actually slightly different phenomena studied using different point of views and methods (see Irvine, 2017).

In fact, Metzinger (2004[2003]) mentions the possibility that the concept of consciousness may be a cluster concept (see Metzinger, 2004[2003], p. 107), and the general research strategy proposed by him is to focus on (necessary and contingent) *features* of consciousness, such as holism, temporal structure, and phenomenal selfhood. Since not all of these features are necessary, there can be different types of consciousness. Still, he suggests that there are at least a few necessary phenomenal properties, so there could be a core concept of consciousness, characterized by these necessary features. If successful, this approach therefore promises to yield a mature science of consciousness.

However, when it comes to consciousness, there is a subtle, but important problem: understanding consciousness in terms of its characteristic features only shifts the challenge from defining one explanatory target to defining many. What is more, there are two general ways to define features of consciousness: either phenomenologically, by describing features in subjective terms; or on subpersonal levels of analysis, by describing features in objective terms. A problem with the first approach is that it is often unreliable or yields imprecise descriptions; a problem with the second is that it is not theory-neutral.

Metzinger tries to overcome these problems by treating descriptions at different levels of analysis as constraints that a fully developed concept of consciousness must respect. This is the MICS. It draws on theories and results from different disciplines, targeted at multiple levels of analysis. Descriptions and assumptions formulated at, for instance, the phenomenological, representationalist, and neurobiological levels of analysis provide different perspectives on the same phenomena, which can ultimately yield necessary and sufficient conditions for applying the concept of phenomenal representation.

An implication of this approach is that there is no methodological separation between specifying the *explanandum* and specifying the *explanans*: Metzinger does not treat the two questions formulated above as independent. Although one of his goals is to take phenomenological reports and intuitions about conscious experience seriously^2^, Metzinger also points out that empirically informed theories of consciousness and subjectivity are likely to be counter-intuitive (see Metzinger, 2004[2003], p. 2), may “destroy […] ‘common-sense appearances’” (Metzinger, 2006, p. 2–3), and that new conceptual tools (such as a differentiated notion of global availability, see Metzinger, 2004[2003], p. 31) may even *change* our phenomenology (see Metzinger, 2006, p. 2)^3^.

I shall not discuss this assumption here. Instead, I will focus on problems and challenges this approach has to face, as well as on possible solutions. To illustrate the approach, I shall describe the method using the example of global availability.

### 2.1. MICS at work: the example of global availability

The general point of departure for Metzinger's MICS is the following: “First, I construct the baselines for a set of criteria or catalog of constraints by which we can decide if a certain representational state is also a conscious state. I propose a multilevel set of constraints for the concept of phenomenal representation.” (Metzinger, 2004[2003], p. 107). I shall now illustrate how this is meant to work by summarizing how Metzinger deals with the constraint of *global availability* (GA). This will also allow us to point to some problematic aspects of the approach. The main problem that will be revealed by this illustration is that MICS, as presented in *Being No One*, is not a theory-neutral approach, and does not specify how to integrate and compare analyses at different levels.

Global availability (GA) is the first constraint discussed by Metzinger in *Being No One*. It is also one of the most important constraints, since it is often treated as a proxy for consciousness (see Metzinger, 2004[2003], p. 117–118). Metzinger does not *identify* phenomenal representations with representations that make their contents globally available, but only specifies GA as the main functional role associated with consciousness (this is closely connected to what has been called the “integration consensus” regarding functions of consciousness, see Morsella, 2005). Conceptually, GA is first and foremost a functional constraint, but Metzinger also analyzes it on the phenomenological, representationalist, informational-computational, and neurobiological levels of analysis. The aim in this section is not to evaluate and discuss these analyses as such, so I will just provide some representative quotations:

[Phenomenological analysis:] In short, global availability is an all-pervasive functional property of my conscious contents, which *itself* I once again subjectively experience, namely, as my own flexibility and autonomy in dealing with these contents. (Metzinger, 2004[2003], p. 118).
[Representationalist analysis:] Phenomenal representational content necessarily is integrated into an overarching, singular, and coherent representation of reality as a whole. (Metzinger, 2004[2003], p. 120).
[Informational-computational analysis:] Phenomenal information is precisely that information directly available to a system in the sense just mentioned. (Metzinger, 2004[2003], p. 120).
[Functional analysis:] [P]henomenal states can interact with a large number of specialized modules in very short periods of time and in a flexible manner. One-step learning and fast *global* updates of the overall reality model now become possible. (Metzinger, 2004[2003], p. 121).
[Neurobiological analysis:] At present hardly anything is known about the neurobiological realization of the function just sketched. […] Among many competing hypotheses, one of the most promising may be Edelman and Tononi's dynamical core theory […]. (Metzinger, 2004[2003], p. 122).

Note that these quotations cannot do justice to Metzinger's analysis of GA, and mainly serve the purpose of illustration. They also serve to highlight a potential general problem with MICS (as described in *Being No One*): Either an analysis at a given level is relatively general (“Phenomenal information is precisely that information directly available”), and hence of little use; or the analysis is more specific, but also more speculative and potentially controversial. We can see this by focusing on the neurobiological level of analysis.

Metzinger mentions Edelman's and Tononi's dynamical core theory (DCT; see Tononi and Edelman 1998; Edelman and Tononi, 2000). This theory (which can be regarded as a precursor of Tononi's integrated information theory (IIT) of consciousness, see Oizumi et al., 2014) posits *functional clusters* in the brain, which can in principle be determined by computing the mathematically defined cluster index of neural populations. The theory operationalizes the concept of neural integration, and this integration may underpin the functional profile associated with GA.

But things are not that simple, as Metzinger notes (Metzinger, 2004[2003], p. 123). One problem is that different types of GA can be distinguished (see Metzinger, 2004[2003], p. 124). If a theory like DCT is regarded as a theory of the neural implementation of GA, this raises the question which type of GA is explained by DCT. Perhaps forming a functional cluster is sufficient for displaying each of the different types of GA? Perhaps it is just a necessary condition on neural correlates of GA? In the worst case, such theories only deliver descriptions of neural implementations, without illuminating *why* certain neural features correlate with features of conscious experience. In general, being able to determine the “degree of fit” between descriptions on different levels of analysis would be desirable. Having established a high degree of fit between descriptions, one could then explore to what extent some of them *explain* the phenomena referred to in the others.

### 2.2. Problems with MICS

However, as things stand, MICS does not provide a rigorous method of specifying relations between phenomena and structures targeted at different levels of description. Taking a multi-level perspective is still useful because it enables a rich characterization of features of consciousness, which are notoriously difficult to define. But even assuming that the result of applying MICS is a set of true propositions about consciousness: What exactly is the relation between the described features and phenomena? How do we decide whether two descriptions just intuitively fit together or whether they actually target *the same* structures on different levels of description?

A related, but more fundamental problem, has been pointed out by Weisberg (2005). He criticizes MICS for failing to make a clean separation between the *explanandum* and the *explanans*, since Metzinger in some cases refers to particular (but controversial) theories, such as DCT, to analyze constraints (see Weisberg, 2005, p. 5–6).

This can only succeed if there is a way to falsify theories or to establish which of two or more competing theories provides the best analysis of a given feature (such as GA). Weisberg is skeptical regarding this, and anticipates that, from the vast number of analyses provided by Metzinger, “competing theorists will likely pick and choose those elements amenable to their view, and disparage the others as extraneous or incorrect. The same unproductive cycle of debate concerning the nature of consciousness will reemerge within Metzinger's MICS structure.” (Weisberg, 2005, p. 5).

Depicted in this way, MICS resembles a loose bundle of theoretical assumptions, empirical hypotheses, and characterizations of consciousness, without providing a rigorous method to integrate and revise them. This is certainly not a completely accurate description of Weisberg's view on MICS, but highlights important aspects of his critical remarks. If we assume that no theory-neutral phenomenological characterization of the *explanandum* is possible (for instance, because there are no first-person data, see Metzinger, 2006, p. 1), descriptions and theories at all levels of description must be used to define (and also explain) the *explanandum*. For instance, this also means that neurobiological data can provide bottom-up constraints (see Lamme, 2010). In principle, these constraints can be continuously refined and thereby yield ever more specific characterizations of the target phenomenon (because the more specific the constraints, the less ways there are to satisfy them).

In practice, however, the very fact that these constraints are not theory-neutral can lead researchers to accept only some of the constraints, while rejecting other constraints. Hence, unless the framework specifies how different theories (which may entail different constraints) can be compared and evaluated, progress gets stuck in the unproductive cycle of debate mentioned by Weisberg.^4^

There is more to be said about MICS—however, instead of continuing with a more thorough and faithful exegesis, I shall now discuss, more generally, how the problem of integrating analyses at different levels can be solved. To this end, I shall first define in more detail what this problem consists in, by formulating two matching problems, and by discussing existing proposals on how to solve these problems (section 3). In the concluding section, I shall briefly discuss a further problem, which consists in integrating analyses of *different* constraints. This is required in order to move from accounting for a bundle of features (that are more or less closely associated with consciousness) to a unified theory of consciousness, which specifies and explains a unique target phenomenon.

## 3. Two matching problems

The fact that MICS analyzes individual constraints on multiple levels calls for an inter-level integration between constraint analyses at different levels. This challenge can be described in terms of the following two problems: the problem of matching descriptions^5^ and the problem of matching predicates^6^. In general, solving these problems will specify how an integrated MICS can yield not just characterizations of target phenomena, but also how it enables explanatory accounts, which is a central goal of the science of consciousness.

### 3.1. The problem of matching descriptions

Different levels of analysis provide very different concepts that may often seem semantically incommensurable: phenomenological reports operate on a personal level of description, neurobiological descriptions on a subpersonal level; phenomenal properties are sometimes described as ineffable, whereas scientific data are the results of measurements and are intersubjectively accessible (cf. Metzinger, 2006, p. 1); phenomenological reports feature contents of consciousness, neurobiological concepts refer to their vehicles. Semantically, this means that it is unclear how to determine whether descriptions on different levels of analysis have the same truth conditions or not. More generally, the question is how to match descriptions at different levels of analysis with one another. This is the problem of matching descriptions. There are different possible solutions to this problem, which specify how to assess the “degree of fit” between descriptions. Here, I will discuss two strategies. The first seeks to establish a logical equivalence between descriptions, the second determines the coherence between descriptions. Each strategy poses further problems and challenges.

#### 3.1.1. Matching descriptions in terms of logical equivalence

At least ideally, it would be desirable to arrive at a multi-level constraint analysis that consists of *logically equivalent* descriptions^7^. In practice, this may seem overly ambitious: If a phenomenological description was logically equivalent to a neurobiological description, this would, arguably, mean that certain neural states necessarily go along with the instantiation of certain phenomenal properties. For if two propositions are logically equivalent, this equivalence holds necessarily. But the assumption that phenomenal truths are logically equivalent to certain physical truths is highly controversial, and most authors explicitly reject it. In fact, this is the core of the *hard problem*: phenomenal properties do not supervene logically on physical properties, so phenomenal truths are not logically entailed by physical truths (see Chalmers, 1995, 1996, p. 93).

But Metzinger's MICS does not presuppose such a controversial assumption. As he points out in *Being No One*, “[t]he primary target of the current investigation, therefore, is ordinary humans in ordinary phases of their waking life” (Metzinger, 2004[2003], p. 14). I interpret this statement as meaning that all constraint analyses are restricted to “ordinary” conscious states, and hold at most with nomological necessity. So when Metzinger stipulates that, functionally, global availability means that phenomenal states “can interact with a large number of specialized modules in very short periods of time and in a flexible manner” (Metzinger, 2004[2003], p. 121), this proposition holds with at most nomological necessity and only for ordinary phenomenal states. Similarly, Metzinger's phenomenological description that “global availability is an all-pervasive functional property of my conscious contents, which *itself* I once again subjectively experience” (Metzinger, 2004[2003], p. 118), holds with the same restrictions. (This is not to deny that a mature science of consciousness must also take phenomenal states in sleep and non-standard states into consideration. In fact, dreaming and disorders of consciousness are among the phenomena discussed in *Being No One*.)

More specifically, such a restricted equivalence statement would have the following form: □∀*x*(*Sx* → [*Px* ↔ *Nx*]), where □ is the modal operator for nomological necessity, *Sx* means “*x* is a human subject of experience in an ordinary phenomenal state”, *Px* stands for a description referring to a phenomenal property (e.g., “*x* instantiates phenomenal property *P*”), and *Nx* stands for a description referring to a neurobiological property (e.g., “*x* instantiates neurobiological property *N*”).

Assuming a restricted equivalence between analyses is not as controversial anymore, but the problem we now have is that the qualified statements include a reference to “ordinary phenomenal states,” which must here be treated as an unanalyzed term. Hence, there is no way of assessing the equivalence assumption by formal considerations. Again, it seems that not having a clear definition of the explanandum (i.e., consciousness) renders the entire project futile.

But things are, of course, not as bad as it seems. We not only have access to descriptions of phenomenal and functional/physical properties, but also to phenomenal states themselves. We can report whenever we are in a phenomenal state that displays a certain phenomenal property and can, in principle, investigate all physical properties instantiated during that time (again, this holds at least for ordinary phenomenal states and features that are not too subtle to be described using conventional means). Hence, the assumption that certain constraint analyses are equivalent (nomologically, and for ordinary phenomenal states) is a hypothesis we can assess *empirically*. This is no new insight, but just a theoretical motivation for the quest for *neural correlates of consciousness* (NCCs) (see Chalmers, 2000; Fink, 2016). If the occurrence of a particular type of phenomenal state (displaying phenomenal property *P*) is strongly positively correlated with the occurrence of a particular type of neural state (displaying neurobiological property *N*), this provides an empirical justification for the claim that a phenomenological constraint analysis (referring to *P*) is equivalent to a neurobiological constraint analysis (referring to *N*), at least restricted to ordinary waking states.

A problem is that empirically measured correlations may be too weak to support an equivalence hypothesis. In fact, this is to be expected, given that analyses are restricted to “ordinary phenomenal states,” which is a vague term. Hence, there will always be borderline cases in which deciding whether a given data set must be included in statistical analyses will require a judgment call. Apart from that, standard research on NCCs only identifies minimally *sufficient* neurobiological properties, because one and the same type of phenomenal state could be underpinned by different neural activity. However, an equivalence statement would require that the neurobiological properties also be *necessary*. So although neural correlates can inform and support constraint analyses, they can at most be part of an integrated constraint analysis. What is more, they fall short of the target of justifying equivalence claims.

#### 3.1.2. Matching descriptions in terms of coherence

Further considerations may suggest that trying to find logically equivalent constraint analyses may be too ambitious. Although nothing may be wrong with this in principle, it presupposes that constraint analyses come in the form of conceptually precise and succinct descriptions. In practice, and at least currently, constraint analyses are often rather tentative and highglight several different aspects of a given constraint. At least the analyses in *Being No One* are intended as preliminary descriptions that can (and must) be refined by future research. So instead of trying to formulate logically equivalent descriptions at different levels of analysis, it may be more viable to formulate sets of descriptions at each level, and trying to match those with one another. Instead of determining whether these sets of descriptions are equivalent, the aim will then be to determine whether they are coherent^8^.

A problem with this strategy is that there are different ways to measure the coherence of statements, and different measures can yield rather different results (see Brössel, 2013, p. 615). In the worst case, deciding which coherence measure to use could be completely arbitrary. In principle, however, there may be good justifications for using a particular measure, so the strategy should not be ignored from the very beginning.

We can connect this strategy to the project of finding correlates of consciousness, as well. The aim will then not be to find a single neural correlate of a given type of phenomenal state, or to associate a given type of neural activity (or mechanism) with a single type of phenomenal state. Rather, the aim will be to associate sets of (similar) types of phenomenal state with types of neural activity. A possible example comes from Metzinger's work on mind-wandering: mind-wandering cannot be associated with a single type of phenomenal state, but rather with a cluster of slightly different types, such as mental planning, periods of insomnia, depressive rumination, and others, which all come with a different phenomenology. Correspondingly, the underlying neural mechanisms cannot be associated with a single neural area, but rather seem to be underpinned by activity that “overlap[s] with activity in the default mode network […], but […] also extends to other functional structures such as the rostrolateral prefrontal cortex, dorsal anterior cingulate cortex, insula, temporopolar cortex, secondary somatosensory cortex, and lingual gyrus” (Metzinger, 2017, p. 11; also see the references cited there).

As already pointed out, one can doubt that the equivalence-strategy and the coherence-strategy can successfully be carried out, without making theoretical assumptions (such as the choice of a particular measure of coherence) that themselves need further justification and are subject to debate. A more fundamental problem is the following: finding correlations or coherences between events or variables picked out using different descriptions is not explanatorily sufficient. What we want to understand is *why* these correlations or coherences exist: we need “explanatory correlates of consciousness (ECCs)”, as Seth and Edelman (2009) have called them. This brings us to the second integration problem: unless the *predicates* involved in theories at different levels can be matched, correlations will in part remain mysterious.

### 3.2. The problem of matching predicates

Even assuming that the problem of matching descriptions can be solved, this only establishes correlations between events or variables picked out using equivalent (or coherent) constraint analyses. It seems that, independently of whether the problem of matching descriptions can be solved, we need a more fine-grained strategy to gain a deeper understanding. This can be achieved by matching predicates.

Similarly to “matching descriptions,” “matching predicates” is a technical term that must be defined. If we restrict constraint analyses to human beings, predicates referring to phenomenal properties and predicates referring to neurobiological properties will be among the predicates that have to be matched. Given this restriction, it seems reasonable to require that two predicates match only if they are co-extensional. However, while this requirement may be necessary, it is not sufficient, for the following reasons:

1. Distinct phenomenological predicates can be co-extensional. This is suggested by the fact that necessary features of consciousness will be displayed by all (ordinary) conscious experiences.
2. Furthermore, since the neural activity underpinning the instantiation of properties of interest is typically distributed, we can expect that many phenomenal properties will be underpinned by at least highly similar activity (involving largely overlapping neural structures), which can make it extremely difficult, in practice, to dissociate neurobiological properties associated with distinct phenomenal properties.
3. Most importantly, the hypothesis that a phenomenological predicate *P* is co-extensional with a neurobiological predicate *N* will most likely be justified by showing that the corresponding phenomenal/neurobiological properties are correlated. This, however, was already suggested above, in the context of matching descriptions. In other words, defining “matching predicates” in terms of correlation does not yield a more fine-grained method of matching, but collapses into the first approach to matching descriptions, discussed in section 3.1.1.

If co-extensionality is not sufficient for matching, what about identity? Arguably, if it could be shown that the predicates used in constraint analyses on different levels are identical, this would strongly suggest a high degree of fit between the analyses. Unfortunately, it seems that identity is too strong a requirement (but see section 3.2.5).

As a reviewer pointed out, it seems that phenomenal properties *cannot* be identical with properties of neural correlates: my brain is not red when I have an experience as of something red. However, “experienced redness” is not the same as “redness”. As the reviewer pointed out, this is what Place (1956) called the *phenomenological fallacy*: “the mistake of supposing that when the subject describes his experience, […] he is describing the literal properties of objects and events on a peculiar sort of internal cinema or television screen” (Place, 1956, p. 49). My brain is not red when I consciously perceive something red, but neither is my conscious experience. My experience has a phenomenal property that allows me to describe what I am seeing as red, but describing this property as “experienced redness” does not entail that it cannot be identical to a neurobiological property. In general, lacking a clear definition of “phenomenal property,” it is impossible to assess whether or not this type of property can be said to be a property of neural processes or structures. Still, demanding that solutions to the problem of matching predicates show how an identity between phenomenological predicates and neurobiological predicates can be established would clearly be too strong. However, solutions should establish more than just co-extensionality (for the three reasons givens above).

Again, there are different strategies to solve this problem, and one is exemplified by the already-mentioned project of finding ECCs, which proceeds by operationalizing phenomenal properties (Seth, 2009). I call this the *head down and charge* approach, because it reduces phenomenological characterizations to a minimum. A further approach consists in using characterizations at an intermediate level of analysis, together with a theory of representational content. I call this the *third man* approach, because it depends on a mediating theory (the label also alludes to the “third man argument” – this emphasizes that the approach posits additional entities, viz., representations, which makes it less elegant). Another approach introduces a formal theory with terms and concepts that can be interpreted at different levels at the same time. I call this the *giga-bingo* approach. Finally, I shall discuss what I call the *metrical* approach.

#### 3.2.1. The *head down and charge* approach to matching predicates

In order to find ECCs, properties of consciousness have to be operationalized, i.e., operational criteria have to be defined to determine whether a given neural process displays the property in question or not (cf. Clowes and Seth, 2008, p. 92). An example given by Seth is the property of complexity (in the sense of being both integrated and differentiated). Operational definitions of complexity (such as Seth's causal density or Tononi's integrated information, see Seth et al., 2011), which measure the compresence of integration and differentiation, can be applied to descriptions of neural dynamics. If a neural process that correlates with consciousness also displays complexity, as defined by an operational criterion, this may give us a hint as to *why* the correlation with consciousness exists. In other words, we may have found an ECC.

An example given by Tononi (2012, p. 293–294) is the difference between the cerebellum and the thalamo-cortical system: the cerebellum is a giant neural structure, and yet activity in the cerebellum does not seem to be sufficient for consciousness. Cortex and thalamus, by contrast, are comparatively small structures and still seem to be sufficient to generate conscious experience. So *why* does activity in one, but not in the other, correlate with consciousness? Tononi's answer is, of course, that the cerebellum is not fit to generate integrated information.

This is only one reason why the integrated information theory (IIT) is a good example of this approach. Another is that, “according to IIT, there is an identity between phenomenological properties of experience and informational/causal properties of physical systems” (Oizumi et al., 2014, p. 3). Two phenomenal properties which the theory seeks to operationalize are, not surprisingly, *information* and *integration*. The theory provides relatively unspecific phenomenological characterizations of these properties, and mostly delegates the task of characterizing the properties at hand to subpersonal levels (which already indicates that the goal of establishing an identity between phenomenal and informational properties should not be taken at face value).^9^ In principle, the adequacy of these operationalizations can be assessed by determining whether neural states in conscious subjects are integrated and informative in the sense specified by the theory's formal definitions. This is particularly promising for candidates for necessary properties of consciousness (cf. Seth, 2009, p. 50).

But this presupposes that we already know whether a given property is a necessary property of consciousness, which arguably requires a (theory-neutral) specification of the explanandum (otherwise, we do not even know which property is supposed to be a necessary property of consciousness; I discuss this point with respect to the unity of consciousness in chapter 3 of Wiese, 2018). Furthermore, this project seems to be restricted to “structural properties,” i.e., quite general properties. We won't be able to explain the experienced redness of a tomato, for instance. Finally, just being able to determine the adequacy of an operationalization *in principle* does not help if, empirically, different operationalizations are either non-independent or do not converge, because then the central question remains: do they really operationalize the same thing or not (for a critical discussion, see Irvine, 2017, p. 100–101)?

#### 3.2.2. The *third man* approach to matching predicates

As we saw in section 2.1, Metzinger (2006) expresses doubts that theory-neutral characterizations of the explananda in consciousness research are possible (to say the least). This can be justified by noting that at least many phenomenological reports do not specify an *explanandum*, but only an *analysandum*, i.e., “a certain way of speaking about a phenomenon, a way that creates logical and intuitive problems.” (Metzinger, 2004[2003], p. 3). The *head down and charge* approach affirms this statement, and seeks to dispense with rich phenomenological descriptions. However, one could argue that an analysandum is only a rough, incomplete, or partly indeterminate characterization of an explanandum, which can be explicated by taking theories from subpersonal levels into account. This is the strategy pursued by the *third man* approach.

An excellent example can be found in research on the phenomenology of time-consciousness^10^. We can consciously perceive temporally extended processes and successions of events. A standard example is the movement of the second hand of a clock: most people would say that we *directly* perceive the second hand's motion, in the sense that we do not need memory or conscious inference to conclude that it is moving. This can be contrasted with the motion of the hour hand of a clock, which we cannot directly perceive (provided it moves continuously), because its motion is too slow. Still, we can become aware of the fact that the hour hand has moved, if we notice that its current position differs from its position a while ago. Crucially, this is different from the way in which we perceive the second hand's motion. The fact that we can directly experience at least some types of change or succession creates the following problem (here described by Robin Le Poidevin):

[I]t seems natural to talk of perceiving one event following another (the thunderclap as following the flash of lightning), though even here there is a difficulty. For what we perceive, we perceive as *present*—as going on right now. Can we perceive a relation between two events without also perceiving the events themselves? If not, then it seems we perceive both events as present, in which case we must perceive them as simultaneous, and so not as successive after all. (Le Poidevin, 2015, first section)

The phenomenological description that we experience successions of events *as present*, but not *as simultaneous* creates a puzzle. For it seems that what happens at the same moment (within the same present) must also happen simultaneously. There are numerous attempts at making sense of such descriptions (for an overview, see Dainton, 2014), and some of them can be considered as instances of the *third man* approach.

A good example is Grush's account of how aspects of Husserl's phenomenology can be linked to research in computational neuroscience (see especially Grush, 2006, 2016). A first idea is that we sometimes experience events *as past* (e.g., when we remember them) or *as present* (e.g., when we perceive them), or *as forthcoming* (e.g., when we imagine or anticipate them), and this is one way in which we can experience events as temporally related. However, there is another way in which we experience events as temporally related, namely, when we experience two or more events as present:

[M]y own theory is restricted in its application to the sub-200 ms scale, and the claim is that *at that scale* […] events are represented as standing in relations of *earlier than, simultaneous with*, and *later than*. (Grush, 2016, p. 8)

So the task Grush sets for himself is to provide a more fine-grained analysis of the puzzling phenomenological description referred to above, and to explain how events can be experienced *as present*, but still as non-simultaneous. Within this task, phenomenological characterizations (such as Husserl's) are only treated as analysanda, and Grush's account can be seen as an attempt at specifying in more detail what the contents of consciousness are.

Here, a second idea comes into play. The idea is that we can analyze contents of consciousness by referring to types of mathematical contents^11^ that figure in computational models in theoretical neuroscience. More specifically, the contents of the experienced present can be matched with the mathematical contents of a *trajectory estimate*. A trajectory estimate represents states of a dynamical system at different times: it contains an estimate of the system's current state (based on sensory signals and an internal model of the system), estimates of the system's previous states (based on previous estimates and current sensory signals), and estimates of the system's anticipated states (based on the estimated current state and an internal model; see Grush, 2005, p. S211).

This analysis brings us closer to an understanding of how two events can be experienced as present, but as non-simultaneous: a trajectory estimate represents events as being temporally related, although none of them is represented as past or as future. This is, of course, only a very brief description of Grush's comprehensive analysis. In order to fully account for the difference between events that are experienced as past and those that are experienced as present, one would, for instance, also have to specify functional differences between representations of present events and representations of past events. Still, even this short description hopefully illustrates the idea that *types* of mathematical contents (trajectory estimates) can help analyze *types* of experienced contents (the contents of the experienced present).

A further illustration can be found in my recently proposed extension of Grush's model, the *hierarchical trajectory estimation model* (Wiese, 2017). Here, the idea is that drawing on another type of mathematical contents, viz. states of a *hierarchy* of dynamical systems (as posited by predictive processing models, see Kiebel et al., 2008), can further help analyze phenomenological descriptions of temporal experience. More specifically, the idea is that contents coded by representations higher in such a hierarchy code contents that can be described as “temporal gist,” which accounts for the continuity of experience. Again, this is only a simplified description of the entire account (for more details, see Wiese, 2017).

As such, analyses of types of experienced contents in terms of types of mathematical contents do not provide a way to determine whether such analyses are adequate or not. For one could object that types of mathematical contents that figure in computational models provide at best *metaphors* for experienced contents: the contents of the experienced present are *like* a trajectory estimate. But how should we determine whether this is a good metaphor?

As Yoshimi (2014, p. 3145) suggests, certain metaphors can help explain phenomenal properties in terms of properties of their neural vehicles. He calls them “bridge metaphors.” These metaphors enable us to construct a mapping from states of the vehicles to conscious states. Based on such a mapping, we can make predictions about the phenomenology (of time consciousness)^12^. For instance, Grush's account predicts that the content of momentary consciousness always contains an anticipation of what will be happening within the next fraction of a second, and he uses research on representational momentum to support this claim (see Grush, 2006, p. 445–446). Predictions can also be about the underlying neural mechanisms. When it comes to time consciousness, one of the questions that are relevant here is which vehicle properties are relevant to the way temporally extended processes or successions of events are experienced (see Wiese, 2017). I will not enter this debate here, but only want to notice that the fact that Grush's account is specific enough as to allow for predictions regarding the phenomenology of time-consciousness, as well as its neural underpinnings, fosters the view that the *third man* approach constitutes a viable way of matching predicates: A mediating theory analyzes types of contents of consciousness in terms of types of mathematical contents, and this analysis can be evaluated by testing predictions about the phenomenological and other levels of analysis.

#### 3.2.3. The giga-bingo approach

The giga-bingo approach posits a theory comprising terms and descriptions that can be interpreted at different levels of analysis. This theory will typically be a formal theory. Such a theory differs from a mediating theory (such as a representationalist theory), because it does not contain two or more classes of terms and descriptions, such that one of them is applicable to phenomenological reports, whereas another is applicable to neural data (or to descriptions at other subpersonal levels). Instead, the hope is to find a single set of concepts that is applicable to *all* levels of analysis at the same time.

The label “giga-bingo approach” is inspired by the *giga-bingo illusion*, a term coined by the Swiss meditation teacher Fred von Allmen. Someone who is under the giga-bingo illusion is confused or dissatisfied with their current situation, but believes that sitting down and meditating once will instantly change everything for the better. Admittedly, this renders the label “giga-bingo approach” tendentious. However, it emphasizes that the giga-bingo approach is more ambitious than the mediating approach. It also comes with several advantages:

(i) If the giga-bingo theory is a formal theory, it will be more metaphysically parsimonious than a representationalist theory. For a representationalist theory posits representations as additional entites, and this already is a controversial posit. A formal theory does not usually presuppose additional entities of this kind. It only offers a re-description of already existing theories and assumptions.

(ii) The giga-bingo approach offers a more elegant solution to the problem of matching descriptions than the third man approach. It does not describe seemingly incommensurable phenomena as properties of a single, third entity. Rather, it describes them as different ways of conceiving one and the same property or structure. The giga-bingo approach is, potentially, also more faithful to phenomenological descriptions than the head down and charge approach, because it does not mainly treat them as heuristically useful, but as analysanda, that will not only be operationalized, but analyzed in (formal) terms that can also be interpreted phenomenologically.

A good example of this approach can be found in Yoshimi (2007, 2011)^13^. Yoshimi discusses the question whether Husserl's phenomenology can be “mathematized”, and shows that Husserl himself used mathematical concepts, in particular concepts that are also used in dynamical systems theory (DST). Since DST is already being applied to neural data, Yoshimi suggests that if

one can define a function which associates possible brain states with possible conscious states, then relations between the dynamics of the brain as described by computational neuroscience and the dynamics of consciousness as described by Husserl can be pursued with mathematical precision. (Yoshimi, 2007, p. 290)

In particular, the dynamics characterizing consciousness and the dynamics characterizing neural mechanisms may turn out to be identical (or highly similar).

Yoshimi (2011) suggests that a supervenience function between the space of possible brain states and the space of possible conscious states (given a particular brain structure) will allow one to derive the structure of consciousness from the structure of brain space. The structure of brain space is constituted by the set of possible neural trajectories. These can, via the supervenience function, be mapped to trajectories in conscious state space, thereby specifying its structure.

An example given by Yoshimi is the following. If biological neural networks partition their state space in such a way that conscious percepts as of different types of faces (say, female vs. male faces) are underpinned by neural states within distinct parts of the partition, then one can predict that when neural activity changes from a state within one subspace (say, corresponding to female faces) to a state within a different subspace (say, corresponding to male faces), conscious states will change from an experience as of a female face to an experience as of a male face. In Yoshimi's words, this means that “the neural category structure predicts a phenomenological category structure” (Yoshimi, 2011, p. 11). Another way of putting it is that some dynamical features of conscious experience can be explained in terms of their neural underpinnings, because the latter share *the same* dynamical features.

So this aspect of the giga-bingo approach, as exemplified by Yoshimi's account, has the potential to solve the problem of matching predicates with respect to dynamical features: if a supervenience function between brain space and conscious space exists, we can not only predict features of conscious space by investigating brain space; we can also understand (dynamical) features of conscious states if the same features are shared by their neural correlates in brain space.

A problem of the giga-bingo approach is related to its scope: it may only solve the problem of matching predicates with respect to some features of consciousness. As we have seen, Yoshimi (2011, p. 1) focuses on dynamic features of consciousness only: “I show how the dynamics of consciousness can be formally derived from the ‘open dynamics’ of neural activity.” That dynamic (or temporal) features of consciousness can be accounted for in terms of dynamics of neural activity is not as controversial as the assumption that the painfulness of pain experiences can be accounted for in terms of properties of brain states. What is more, it is far from clear that the giga-bingo approach is apt to address such more specific questions, because it is unclear how to associate a given phenomenal property with a mathematical structure.

To be fair, dynamic features are not the only type of features considered by Yoshimi. In Yoshimi (2007), he gives further examples:

For example, Husserl describes experienced space as a conjunction of two manifolds—a “linear manifold of receding,” and a “cyclical manifold of turning” (Husserl, 1997, p. 216). We have also seen that Husserl thought of some manifolds of possibility as having metrical structure, insofar as he takes “distances” between perceptual acts to be meaningful (Husserl, 1973, p. 735). (Yoshimi, 2007, p. 288; citation style adapted)

The conceptual parallels between DST and Husserl's phenomenology are striking, but it is not clear in which way the structures mentioned by Husserl are systematically related to, say, neural structures. So at least in its current form, it seems that Yoshimi's giga-bingo approach cannot solve the problem of matching predicates.

#### 3.2.4. The metrical approach

The metrical approach approach seeks to identify structures displayed by conscious experience (and its neural underpinnings). Since structures can be defined by relations, we can ask: what are relations that structure conscious experience? There are many *experienced relations* we find in ordinary conscious experience, for instance, experienced spatial relations (and many others, see Hill, 1991). These relations characterize structures we find in individual conscious experiences. Considering, more generally, the space of all possible conscious experiences, we can also say that this space as such has a certain structure, endowed by a similarity relation. Here, a similarity relation is a reflexive, symmetric, but not necessarily transitive relation. Intuitively, many conscious experiences are subjectively similar to one another (a pain experience in my left hand can be similar to a pain experience in my right hand), whereas others are relatively dissimilar (a pain experience is not usually similar to an orgasm).

As Rudolf Carnap (1928) pointed out, such a structural description seems to be fundamentally incomplete: it only tells us something about the relations between different entities, but not about the entities themselves. Applied to the domain of consciousness, it seems that a structural description cannot characterize the intrinsic properties of conscious experiences (cf. Chalmers, 1996, p. 235). But these intrinsic properties (i.e., qualia) seem to be what makes conscious experiences special. Hence, it seems that a structural description cannot capture what is special about consciousness.

However, even if conscious experiences are initially treated as indecomposable, and only relations between conscious experiences are considered, such a structural description can be used to define properties of individual conscious experiences. Carnap calls this method *quasianalysis* (see Carnap, 1928, p. 8). In effect, quasianalysis is an extensional way of defining properties. As such, it still has certain limitations. For instance, two systems can have different properties, but the same similarity structures (where a similarity structure consists of a non-empty set with a reflexive, symmetric relation defined on that set). However, the gravity of this limitation can only be assessed by considering concrete examples. As Hannes Leitgeb points out:

If we restricted ourselves to property structures which are determined by similarity structures, quasianalysis would always yield adequate results. The philosophical importance of this fact is that if similarity were in some sense prior to properties […] quasianalysis would necessarily deliver adequate results. Note that […] no two distinct similarity structures can determine the same property structure. On the other hand, two distinct property structures might determine the same similarity structure (Leitgeb, 2007, p. 199)

Of course, many have the intuition that for consciousness, similarity is not prior to properties: if phenomenal properties are the “atoms of consciousness” they are absolutely fundamental. I do not endorse this assumption^14^, but will remain neutral on this point in what follows. For even if we grant that some phenomenal properties cannot be accounted for in terms of structures, it may still be that considering consciousness as being endowed with a similarity structure tells us more about phenomenal properties than we might expect. After all, phenomenal similarity is at least a concept that can be operationalized: if a subject cannot distinguish two perceived stimuli, this means the corresponding conscious experiences are (all other things being equal) more similar to each other than conscious experiences corresponding to two distinguishable stimuli. In fact, subjective indistinguishability, or global indiscriminability (as Clark, 1996, calls it) is a reflexive and symmetric relation, i.e., a similarity relation. This operationalized similarity relation can therefore be used to investigate the structure of (perceptual) consciousness.

The basic idea has roots in classic works in psychophysics. After briefly reviewing these roots, I shall describe the role they play in David Rosenthal's quality space theory (QST). This will allow us to evaluate to what extent an operationalized similarity relation can be used to define phenomenal properties.

Gustav Theodor Fechner (1860) made important breakthroughs on the problem of measuring conscious percepts. Following his teacher Ernst Heinrich Weber, Fechner realized that a conscious percept itself cannot be measured. For instance, there is no reliable way of measuring the (experienced) intensity of a light sensation as such. However, one can measure by how much a given stimulus has to be changed in order to create another sensation that is (just) noticeably different from the first sensation. Just noticeable differences can function as units, which allow one to measure how different two sensation are. Furthermore, they constitute a method of determining lawful relations between features of stimuli (such as the brightness of a visual stimulus) and experienced features of conscious percepts (such as the experienced brightness of a visual percept)^15^.

Fechner's psychophysics has been an influential approach in empirical psychology; further milestones in its development include works by von Helmholtz (in the nineteenth century), as well as Wright and MacAdam (in the middle of the twentieth century; for an excellent historical and systematic overview, see Isaac, 2013). A fairly recent application of the basic idea is David Rosenthal's QST.

Similarly to psychophysics, this theory starts from the assumption that mental qualities can be characterized by their role in perception. According to Rosenthal (2015), this role consists in enabling discriminations. Hence, mental qualitites can be characterized by investigating which stimuli a subject can distinguish and which it cannot. As a result, a quality space (QS) of perceptual stimuli can be constructed, in which two stimuli are close to each other when the difference between them is a just noticeable difference (JND). Since stimuli are (or are not) discriminable *in virtue of* the mental qualities of the resulting perceptual states, the structure of QS also captures the structure of perceptual conscious experience (Rosenthal, 2015, p. 38). Rosenthal argues that QST can be used to distinguish between different sense-modalities, without having to rely on phenomenological characterizations of what it is like to perceive different types of stimuli (cf. Rosenthal, 2015, p. 50).^16^. QST can most readily be applied to visual perception, but in principle, it can also be applied to other modalities (including olfaction, see Young et al., 2014).

A notable limitation of this approach is that the structure of QS cannot be used to define phenomenal properties (qualia). For the mental qualities in virtue of which stimuli are discriminated are assumed to be the same for conscious and unconscious perception (see Rosenthal, 2015, p. 34). Therefore, QST does not tell us what is special about conscious perception. It does not tell us how the structure of conscious perception differs from the structure of unconscious perception.

Or so it may seem. It may be the case that some discriminations can only be made consciously, or that we fail to correctly discriminate certain stimuli consciously, although we would have been able to discriminate them unconsciously. These are speculative suggestions, but they draw attention to the fact that some structural features of conscious perception may be necessary features of consciousness.

We can illustrate this by considering the two constraints on the concept of consciousness that Metzinger identifies as necessary in *Being No One*: These two constraints are *global availability* (GA) and *activation within a window of presence* (AWP) (see Metzinger, 2004[2003], p. 136). Although GA is primarily a functional constraint, Metzinger also associates it with phenomenal properties:

[T]here is a globality component and an availability component, the latter possessing a phenomenological reading in terms of autonomy, flexibility, and selectivity of conscious access to the world. […]
This is what constitutes the phenomenological reading of “globality”: being an integral part of a single, unified world. (Metzinger, 2004[2003], p. 119-120)

If this is correct, and if GA is necessary for consciousness, then the experience of being part of two disunified worlds is impossible. Note that this does not necessarily involve experiencing *oneself* as an integral part of a world. It may be possible to spell this out in terms of perceived events. For instance, while it is possible to experience two different sounds as coming from different directions, it is probably impossible to experience two sounds as coming from two different worlds.

AWP entails that conscious experience necessarily goes along with a phenomenal moment, a *Now* that subjectively differentiates present events from future or past events:

There are temporal gestalts, islands of individually characterized Nows, but the background against which these islands are segregated is itself not static: it possesses a direction. Subjective time flows from past to future, while at the same time allowing us to rise above this flow in the immediacy of the conscious presence. (Metzinger, 2004[2003], p. 126–127)

Crucially, the subjective flow of time has a direction that cannot be reversed. Although subjects in altered states of consciousness may report that time seems to slow down, stop, or become “gappy” (Wittmann, 2015; Berkovich-Ohana and Wittmann, 2017), it may be impossible to experience a reversal of time (cf. Riemer, 2015): this would correspond to experiencing events as unfolding from future to past.

If GA and AWP are logically necessary constraints on conscious experience, they entail that certain conscious experiences are logically impossible. More specifically, although it may be logically possible to represent certain contents non-phenomenally (such as “I am perceiving sounds from two distinct worlds,” or “I am experiencing time as unfolding from future to past.”) it may be logically impossible to represent such contents phenomenally.

The hypothesis that there are logically necessary phenomenological constraints entails that there are asymmetries in the structure of the space of logically possible conscious states that are related to the difference between conscious and unconscious states. If these asymmetries can be accounted for in terms of properties identified on subpersonal levels of descriptions, it should be possible to find the same asymmetries in the structures of functional, informational-computational, and neural state spaces that underpin conscious experiences.

GA and AWP are very general constraints, and it is possible to refine them by, for instance, distinguishing different types of GA (see Metzinger, 2004[2003], p. 124). So even if GA and AWP were the only necessary constraints on consciousness, this would not mean that there are only two phenomenological constraints on consciousness. Future research should explore the range of possible conscious states in more detail, to find candidates for asymmetries in the structure of consciousness.

#### 3.2.5. Summary: how do the approaches discussed solve the problem of matching predicates?

How do the approaches discussed define “matching predicates?” The *head down and charge* approach takes phenomenological characterizations as a heuristic starting point to develop operational definitions. It predicts that the properties thus defined are actually displayed by neural activity or structures. If the prediction is corroborated, this supports the assumption that the operationalization is adequate and that the phenomenological predicate matches the predicate referring to the operationalization.

The *third man* approach analyzes types of phenomenal content in terms of types of mathematical content, specified by computational models. From such analyses, predictions about phenomenal contents, as well as about neurobiological mechanisms (implementing the computational models) can be derived. There is a match between the analyzed phenomenological predicates and the predicates referring to mathematical contents/neurobiological mechanisms to the extent that the predictions are corroborated.

The *giga-bingo* approach uses a single (formal) theory that can be interpreted on at least two levels (viz., the phenomenological and the neurobiological levels). There is a match between the predicates unified by the giga-bingo theory to the extent that it allows predicting phenomenal properties from neural properties (e.g., by deriving phenomenal dynamics from neuronal dynamics).

The *metrical* approach is more indirect than the others, but also more ambitious. It identifies *structural* properties. Phenomenologically, these are defined by the relation of experienced similarity (or global indiscriminability, see Clark, 1996). Subpersonally, they can be defined by relations between computational, functional, or neurobiological states. Properties of interest are characterized by asymmetries, and it is an open question whether there are any asymmetries that are only induced by *experienced* similarity relations. This is a (potential) major drawback of this approach; its virtue is that it yields a particularly strong notion of matching: predicates match according to the metrical approach just in case they are *identical*.

## 4. Conclusion and outlook

There are still major hurdles on the way to a mature science of consciousness. We have seen that existing solutions to the two matching problems work at least for some features of consciousness (and at least in principle). In practice, no single approach will succeed on its own, but must be complemented by other approaches. On the upside, this renders the problem of not having a theory-neutral approach less severe—for if different, independently motivated approaches to solving the matching problems converge on the same results, this provides further support for theoretical assumptions made by the individual approaches.

But a fundamental problem remains: even if we arrive at integrated, multi-level accounts of individual features of consciousness, it may still be that “consciousness” is not a unitary concept. In other words, even integrated constraint analyses will not, by themselves, deliver an uncontroversial, universally accepted definition of the explanandum. Is there any hope? I am convinced that defining and explaining consciousness will not forever remain a controversial or arbitrary task. All we need is a single theory that accounts not just for individual features of consciousness, but for all of them (or at least for the most central ones).

To illustrate, Tononi's IIT is a theory that, if correct, primarily accounts for the level of consciousness. In principle, it can also account for phenomenal properties, in terms of shapes in a high-dimensional qualia space (see Tononi and Koch, 2015, p. 12). However, to the best of my knowledge, no one has ever specified the characteristics of those shapes in qualia space that correspond to states that are globally available within a system, are activated within a window of presence, or that are phenomenally transparent. If the theory is correct, there should be a characteristic difference between states that have these features and states that do not. A further step would be to show that states associated with high levels of integrated information necessarily go along with certain shapes in qualia space. This would support the view that consciousness can be defined scientifically (as integrated information), and that it has certain features (namely those features entailed by states with high levels of integrated information). I have my doubts that the required extension of IIT is forthcoming, but future developments may always bring surprising news (for some interesting recent ideas with respect to IIT, see Tsuchiya et al., 2016b).

A further example is the free-energy principle, together with the framework of active inference, developed by Friston et al. (Friston, 2009). Neither the free-energy principle nor active inference are theories of consciousness. However, the concepts and ideas provided by them are being used to analyze an increasing number of features associated with consciousness: experienced objecthood (Seth, 2014, 2015a,b), affective value (Van De Cruys, 2017), phenomenal unity (Wiese, 2018), mental agency (Metzinger, 2017), phenomenal selfhood (Limanowski and Blankenburg, 2013; Hohwy and Michael, 2017; Letheby and Gerrans, 2017), auditory hallucinations (Wilkinson and Bell, 2016), the continuity of conscious perception (Wiese, 2017), phenomenal transparency (Limanowski and Friston, 2018), working memory (Parr and Friston, 2017), and many others. Clearly, just minimizing free energy or prediction error cannot be identical to the computational processes underpinning conscious experience, because that would render virtually all creatures conscious, and many conscious states would not display any features commonly associated with consciousness. However, Friston has recently proposed to define conscious processing as the (temporally thick) minimization of *expected* free energy (for an explanation and more details, see Friston, 2018). If this proposal is on the right track, we have

1. a single computational principle that can be associated with consciousness (viz., minimizing expected free energy) and
2. a principled way of determining which features of consciousness are entailed by this principle and which are not.

For the second point, a computational analysis of multiple features of consciousness is required—and this is exactly what we are witnessing in recent years. If it can be shown that some of these features are entailed by minimizing expected free energy, we get not just an account of a bundle of features, but something that can then be developed into a mature, scientific theory of consciousness.

These remarks are still speculative, but I hope that they serve to illustrate how a mature science of consciousness may be possible. Thomas Metzinger's MICS sets up a framework within which multi-level analyses of features of consciousness can be developed. Various existing approaches show how analyses on different levels can be integrated (by matching descriptions and by matching predicates), and a unitary (core) concept of consciousness can be defined with the help of a unifying theory (which mainly operates at a single level of analysis, but integrates analyses of different constraints).

Ideally, the result will not be yet another theory of consciousness, but will also specify which aspects of previously proposed theories were on the right track and which were not. For instance, global availability (as in global workspace theories, cf. Baars, 1988; Dehaene and Changeux, 2011) or a model of attention (as in attention schema theory, cf. Graziano and Webb, 2015) may well be entailed by a relatively general computational principle, as well as various types of meta-representational processes (as in higher-order theories, cf. Gennaro, 2004). The functions and mechanisms proposed by these theories are not incompatible, and so all we may need is an independently motivated theory that shows how to put the pieces together (cf. Wiese, 2018, p. 230).

## Author contributions

The author confirms being the sole contributor of this work and approved it for publication.

### Conflict of interest statement

The author declares that the research was conducted in the absence of any commercial or financial relationships that could be construed as a potential conflict of interest.

## Footnotes

^1^Seth (2016) calls the problem of accounting for features of consciousness “the real problem” (as opposed to the hard problem).

^2^See Metzinger (2004[2003], p. 2, 136, 198, 455, 505, 576, 591, 608, 611).

^3^Apart from this, the general strategy of letting research projects targeted at different levels of analysis inform one another is common ground between Metzinger's MICS and neurophenomenological approaches, which try to naturalize phenomenology by combining it with neuroscientific and psychological results (cf. Flanagan, 1993; Mangan, 1993; Varela, 1996; Gallagher, 1997; Yoshimi, 2016). More generally, MICS can be regarded as a strategy to develop what Bechtel (1988, p. 101–102) calls an “interfield theory”.

^4^As a reviewer pointed out, empirical progress on consciousness will yield more and more specific bottom-up constraints, making it ever harder to stick to only one's favorite theoretical assumptions, while neglecting those that do not. This is certainly true, but there is no guarantee that the evidence will converge on any particular set of assumptions. The problem is different from the general problem of empirical underdetermination of scientific theories, because the lack of a generally accepted definition of consciousness makes it more difficult to falsify theoretical assumptions about consciousness (cf. Irvine, 2017, p. 100–101).

^5^I am grateful to Thomas Raleigh for suggesting to change the label I used in an earlier version of this paper.

^6^I am grateful to Beate Krickel for suggesting to change the label I used in an earlier version of this paper.

^7^I am grateful to Philip Krekel for suggesting to think about matching in terms of logical equivalence.

^8^I am grateful to Peter Brössel for suggesting to think about matching in terms of coherence.

^9^As an illustration, consider the following phenomenological characterizations: “Consciousness is informative: each experience differs in its particular way from other possible experiences. […] Consciousness is integrated: each experience is (strongly) irreducible to non-interdependent components. […] [S]eeing a red triangle is irreducible to seeing a triangle but no red color, plus a red patch but no triangle.” (Oizumi et al., 2014, p. 2–3). Here are the corresponding operational definitions: “Information: A mechanism can contribute to consciousness only if […] it constrains the states of a system that can be its possible causes and effects – its cause-effect repertoire. […] Integration: A mechanism can contribute to consciousness only if it specifies a cause-effect repertoire (information) that is irreducible to independent components.” (Oizumi et al., 2014, p. 3). The extent to which the state of a system constrains its possible cause-effect repertoire, and the extent to which the cause-effect repertoire is irreducible can be measured using the formal definitions provided by the theory.

^10^A more general example can be found in Williford (2017). Williford suggests that the Bayesian brain paradigm can function as a mediating theory between phenomenology and neurobiology (see Williford, 2017, p. 518).

^11^In speaking of mathematical contents, I here allude to Egan's (2014) distinction between mathematical and cognitive contents. Mathematical contents are the contents specified by a computational model that describes various mathematical functions that are computed. More specifically, mathematical contents are the arguments and values of mathematical functions. Cognitive contents figure in personal-level descriptions of the features and behaviors that are to be explained by computational models.

^12^Yoshimi only suggests that we can use such a mapping to “to induce phenomenological structures” (Yoshimi, 2014, p. 3145) that can then be evaluated by determining whether they cohere with existing phenomenological descriptions, such as Husserl's.

^13^Another example is implicit in Wiese and Metzinger (2012). There, the idea was that a formal mereotopological theory could provide axioms and definitions that are interpretable at different levels of analysis, thereby integrating findings at different levels. The main problem with this approach is that the same formal description can have very different meanings when interpreted at different levels of analysis. Worse, it is not always clear whether interpreting a mereological axiom on the phenomenological level even makes sense. For instance, while there arguably is a difference between two different representations and a fusion of these representations, it is unclear that there is a phenomenal difference between two different conscious experiences (had by the same subject at the same time) and a single conscious experience, constituted by the first two experiences (see Wiese, 2018, Ch. 3).

^14^I agree with David Rosenthal (2015, p. 42) on this point: “What it's like for one to be in a particular qualitative state tells us nothing about what type of state it is independent of other comparable states.”

^15^In fact, Fechner proposed three methods to determine such lawful relations, of which the method of just-noticeable differences (*Methode der eben merklichen Unterschiede*) is only one (see Fechner, 1860, p. 71).

^16^For further discussion of this and potentially problematic cases of inter-modal fusion, see Rosenthal (2015, section 1.3).
